# Comparing CD10 Expression With the Clinicopathological Features and Hormone Status of Invasive Breast Cancer

**DOI:** 10.7759/cureus.69836

**Published:** 2024-09-21

**Authors:** Aravindan Kumaravel, Muthuvel Esakki

**Affiliations:** 1 Pathology, Saveetha Medical College and Hospital, Saveetha Institute of Medical and Technical Sciences (SIMATS) Saveetha University, Chennai, IND

**Keywords:** cd10 marker, immunohistochemistry(ihc), invasive ductal cell carcinoma, prognosis, stromal cd10

## Abstract

Background

Worldwide, female breast cancer is the most common cancer (11.7%), followed by lung (11.4%), colorectal, prostate, and stomach. Breast cancer is the fifth most common cause of cancer-related mortality, with lung cancer being the leading cause. In India, breast and cervical cancers are the two most common cancers among women. This study was undertaken to analyse the expression of CD10 in invasive duct cancer (IDC) and its correlation with the various clinicopathological features and hormone status.

Materials and methods

This study was conducted in the Department of Pathology, Saveetha Medical College and Hospital on 42 cases of invasive ductal carcinoma - no special type (IDC NST). The clinical and histopathologic parameters such as age, tumor site, tumor size, histologic type, histologic grade, lymph node metastases, lymphovascular invasion, and perineural invasion were assessed in hematoxylin and eosin-stained sections of the tumor tissue along with the hormone status of positivity for ER, PR and Her2Neu. These parameters were subsequently compared with the expression of CD10 in the corresponding slides and statsitical correlation was done using the chi square test.

Results

The most common age group was more than 40 years, with 41-50 years, and 51-60 years in particular. CD10 was positive in 93% of cases. There was a positive correlation between CD 10 expression and lymphovascular invasion in the study (p-0.006). There was no significant relationship between hormone status and CD10 expression.

Conclusion

A significant association was seen between CD10 expression and lymphovascular invasion. No relation was found between CD10 and the other parameters such as tumour grade, lymph node metastases, lymphovascular invasion, perineural invasion and hormone status. Further studies are required to explore the potential of CD10 as a prognostic marker for IDC.

## Introduction

Breast masses are discrete, small swellings that feel different from the surrounding breast tissue. They are an indication or symptom of several different illnesses. It is critical that women with breast lumps receive the proper evaluation because about 10% of breast masses result in a breast cancer diagnosis [1,2]. Breast carcinoma is the most common malignancy that affects Indian women [3]. The incidence, which ranges from 19 to 33%, is rising annually [4]. Over the past 20 years, India's cancer incidence has shown an "AGE SHIFT," rising from 7% to 15% in the 30 to 40 year age group [5]. 

Cancer of the breast is the most prevalent in India, and up to one-third of people succumb to the disease [3]. Even with effective screening modalities, there are a few subtypes that are diagnosed only at the time of distant metastasis. Immunohistochemistries (IHCs) are crucial for the confirmation of the diagnosis of invasive breast cancer, even though histomorphological features are the primary means of diagnosis [6].

The prognosis of breast cancer is directly linked to the histopathological features such as size of the tumour, grade of the tumour, presence of lymph node metastasis, lymphovascular invasion, perineural invasion and most importantly the hormone status of the tumour. Estrogen receptor (ER), progesterone receptor (PR), and human epidermal growth factor receptor 2 (Her2Neu) are the three widely used biomarkers for analysing the prognosis of breast carcinoma. ER/PR positivity confers a good prognosis whereas Her2 positivity indicates a poorer prognosis. The third subtype in which all three markers are negative, aptly called triple-negative breast cancer, has the worst prognosis with a very poor survival rate [7].

The stromal microenvironment plays an important role in the proliferation of neoplastic cells as well as their distant metastasis [8,9]. Numerous substances produced by stromal cells or neoplastic cells interfere with the interaction between epithelial and stromal cells [10]. Matrix metalloproteinase, produced by stromal cells, plays a crucial role in tumor invasion and metastasis. Studying the role of stromal components may help in developing new treatments for neoplastic conditions [11]. Because they catalyze the cleavage of proteins found in the extracellular matrix, matrix metalloproteinases are essential for the remodeling of tissues.

Neprilysin, or CD10, is a tissue marker used in acute lymphoblastic leukemia/lymphoma. It is a prototype of matrix metalloproteinase (MMP). It is a zinc-dependent metalloproteinase that is expressed on the surface of stromal cells and is elevated in cancerous cells [12]. In the breast, CD10 functions as a stem cell regulator to stop unchecked stem cell proliferation. The signaling factors that drive early common progenitor cells to differentiate into epithelial and myoepithelial cells are inhibited by CD10 [13,14]. Neutrophils, other epithelial cells, lymphoid stem cells, and breast myoepithelial cells all express it. Additionally, the stroma of colorectal, lung, and prostate cancers express CD10 [15]. CD10 is gaining importance recently in tumours like renal cell carcinoma, endometrial stromal sarcoma, and the canalicular pattern of hepatocellular carcinoma [16,17]. The aim of this study is to find out the correlation between CD10 expression and the various clinicopathological features of invasive ductal carcinoma (IDC). This study also aims to find any correlation between CD10 expression and the hormone status of IDC, thus exploring the prognostic potential of CD10.

## Materials and methods

The study was retrospective and was done at the Department of Pathology, Saveetha Medical College, Chennai, after obtaining the necessary approval from the Institutional Review Board. The demographic details were obtained from the registers of the Department of Pathology. Hematoxylin & Eosin (H&E) stained slides and paraffin blocks from all cases of invasive breast carcinomas diagnosed over a five-year period (2019-2023) were accessed. The hematoxylin & eosin slides were reviewed, and the histological features were studied. The hormone status of the cases in the study was obtained from the previous records which used the Allred scoring for ER/PR status and Her2neu scoring was done using ASCO/CAP (American Society of Clinical Oncology/College of American Pathologists) guidelines [18,19]. Appropriate blocks were selected for immunohistochemistry for CD10 staining. Tissue blocks found unsuitable for sectioning due to physical disintegration were rejected and the final sample size was 42.

Inclusion criteria

All histomorphologically confirmed cases of invasive breast carcinoma, no special type from resected mastectomy specimens were included in this study.

Exclusion criteria

Other histological subtypes of invasive breast carcinomas, non-invasive breast carcinomas and tissue blocks found unsuitable for sectioning due to physical disintegration were excluded.

Immunohistochemical staining process

Four micron thick sections were cut from each of the study blocks and mounted on positively charged slides. The slides were subsequently appropriately labelled and incubated overnight at 37^o ^Celsius. The slides were then deparaffinized with xylene and hydrated using decreasing grades of isopropyl alcohol. Antigen retrieval was done by pressure cooker method using Tris - EDTA (ethylenediaminetetraacetic acid) retrieval solution at a pH of 9.0.

The primary antibody used was the Rabbit monoclonal Antibody (NEPP clone) of CD10. The secondary antibody was the PolyExcel Horseradish Peroxidase (HRP)/3,3'-diaminobenzidine​​​​​​​ (DAB) Detection System (PathnSitu Biotechnologies, Secunderabad, India). Benign tonsil tissue was used as the positive control to standardize CD10 staining.

The slides were incubated with the primary antibody (1:100 dilution) followed by the primary antibody amplifier and Master HRP polymer. The chromogen used was the DAB working solution. The slides were then counterstained with hematoxylin and finally, after dehydration and clearing the sections were mounted using DPX (dibutylphthalate polystyrene xylene) mountant.

**Figure 1:**
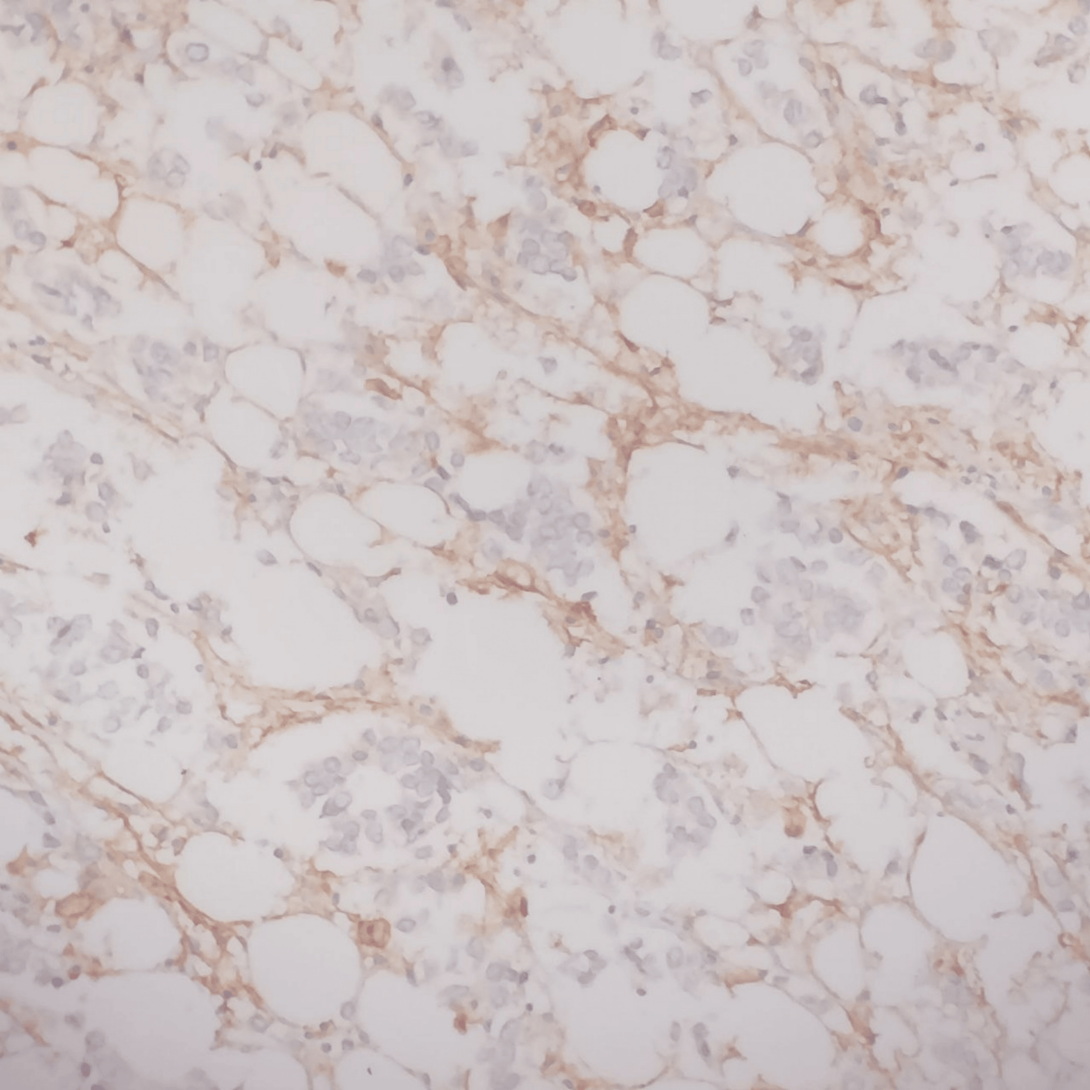
Stromal cells showing both cytoplasm and membrane positivity for CD10. Immunohistochemistry, 40x Objective.

CD10 scoring was done as per the following Table 1 [20]. The pattern of staining for CD10 is both cytoplasmic and membranous positivity in stromal cells (Figure 1). Both negative and weak expression were considered as negative and only strong CD10 expression was considered as positive for statistical purposes.

**Table 1:** CD10 scoring system used. CD10 is considered positive in the stromal cells when both the cytoplasm and membrane take up the stain [20].

Score	Result	CD10 staining
0	Negative	<10% stromal positive cells (cytoplasmic and membrane positivity)
1	Weak	10%-30% stromal positive cells
2	Strong	>30% stromal positive cells

Statistical analysis

Data was entered in a Microsoft Office Excel sheet (Microsoft Corporation, Redmond, USA) and statistical analysis was done with SPSS version 27.0 software (IBM Corp., Armonk, USA). Categorical variables were expressed as frequencies and percentages. When a categorical variable is associated with another categorical variable, the variables are represented in tables. Nominal categorical data between the groups were compared using the Chi-square test. For all statistical tests, a p< 0.05 was taken to indicate a significant correlation.

## Results

In our study population of 42 women with confirmed invasive carcinoma, the most common age group was more than 40 years, with 41-50 years & 51-60 years in particular as seen in Table 2.

**Table 2:** Age distribution of the cases in study

Age in years	No of patients (n)	Percentage (%)
Less than 40	2	5%
41- 50	13	31%
51- 60	10	24%
61- 70	8	19%
More than 70	9	21%
Total cases	42	

Coming to the hormone status, among the cases studied, 18 were positive for ER/PR, 14 were Her2Neu positive, and 10 cases were triple-negative, i.e., negative for ER, PR, and Her2Neu (Table 3). 

**Table 3:** Hormone status of the cases studied ER: estrogen receptor; PR: progesterone receptor; Her2Neu: human epidermal growth factor receptor 2

Hormone status	Number of patients (n)	Percentage (%)
ER/PR positive	18	43%
Her2Neu positive	14	33%
Triple negative	10	24%

CD10 was positive in 93% of the cases (n=39) and three cases were negative. Both CD10 and ER/PR were positive in 16 cases. All 14 cases with Her2Neu positivity also had positive CD10 expression and out of the triple-negative cases, nine were CD10 positive (Table 4). No significant correlation was found between the hormone status and CD10 expression (p=0.443) in this study.

**Table 4:** Association of CD10 Expression with hormone status p=0.443. Chi-squared test. ER: estrogen receptor; PR: progesterone receptor; Her2Neu: human epidermal growth factor receptor 2

CD10	ER/PR positive (n)	Her2Neu positive (n)	Triple Negative (n)
Positive	16	14	9
Negative	2	0	1

Out of the cases in study the tumour was left sided in 52% (n=22) and right sided in 48% (n=20) cases (Table 5). The most common quadrant involved was the upper outer quadrant, with 46% (n=19) of cases showing a tumour there, followed by the lower outer quadrant, which was involved in 21% (n=8) of the cases in study (Table 6). 66% (n=28) of the carcinomas studied were grade II, 19% (n=8) were grade III and the rest 15% (n=6) were grade I according to the Nottingham grading system (Table 7).

**Table 5:** Tumour laterality of the cases

Tumour laterality	Number of patients (n)	Percentage (%)
Left breast	22	52
Right breast	20	48

**Table 6:** Tumour site/quadrant invloved in the cases

Tumour site/quadrant involved	Number of patients (n)	Percentage (%)
Upper outer	19	46%
Lower outer	8	21%
Others	15	33%

**Table 7:** Grades of the tumors in the study

Tumour grade	Number of patients (n)	Percentage (%)
Grade I	6	15%
Grade II	28	66%
Grade III	8	19%

Lymph node metastasis was seen in 62% (n=26) of the cases (Table 8). Lymphovascular invasion was seen in 96% (n=40) and perineural invasion was seen in 46% (n=19) of the cases (Table 9). 

**Table 8:** Lymph node involvement in the cases

Lymph node metastases	Number of patients (n)	Percentage (%)
Present	26	62%
Absent	16	38%

**Table 9:** Cases showing lymphovascular or perineural invasion invasion.

Type of invasion	Number of patients (n)	Percentage (%)
Lymphovascular invasion	40	96%
Perineural invasion	19	46%

The parameters like tumor grade (p=0.118), stage (p=0.702), lymph node invasion (p=0.498), and perineural invasion (p=0.754) showed no correlation with CD10 expression. However, we found a positive correlation between CD10 expression and lymphovascular invasion with a p-value of 0.006.

## Discussion

According to Sung et al., breast cancer is the leading cause of cancer-related death for women in developing nations [21]. The variability in how the disease progresses with breast carcinoma is a challenge for clinicians with regard to tailoring the best treatment based on outcome prediction. Prognostic and predictive biomarkers could play a vital role in accomplishing this task. 

Although breast carcinoma is an epithelial neoplasia, the stromal environment acts as an essential player in breast carcinoma evolution and prognosis. Numerous substances secreted directly by neoplastic or stromal cells under the influence of neoplastic cells, alter the communication between the normal epithelium and stroma [22]. One such vital factor is matrix metalloproteinase (MMP), which acts as a significant player in tumor invasion and metastasis [23]. CD10 is considered the prototype of MMP and has been associated with the aggressive behavior of many tumors [20].

CD10 was positive in 39 patients among 42 patients in our study population. These results are consistent with other studies done by Makretsov et al. and Taghizadeh-Kermani et al., which reported higher percentages of CD10 positivity. The cases positive for CD10 in these studies ranged from 70 to 80% [20,24].

We further correlated all factors that influence the outcome of malignancy with the marker in the study. To start with, there was no significant relationship between hormone status and CD10 expression, with a p-value of 0.443. This result was supported by the findings of Makretsov et al., Jana et al., and Dhande et al., who found no evidence of a significant relationship between CD10 and PR expression [20,25,26]. The results of Kim et al. and Sadaka et al., however, demonstrated a strong inverse connection between the levels of CD10 expression and ER and PR expression [27,28]. Also there was no association between CD10 and Her2Neu in our study, which is in concordance with those reported by Makretsov et al. [20].

Next, moving on to Nottingham’s grade of the tumors, though all cases in grade three were CD10 positive, there was no statistically significant relationship, with a p-value of 0.114. In agreement with our findings, Vo et al. reported that there was no statistically significant relation between CD10 expression and different tumor grades [29]. Coming to relation between staging and CD10 expression, there was no significant relation. The study by Ali et al. showed the same results, including the presence of a significant association between CD10 and lymph node status and an insignificant relation between CD10 and tumor size and tumor stage [30].

In our study, lymph node involvement is not significantly related to the expression of CD10. Dhande et al., however contradicted our study as they showed a relation between CD10 and the status of lymph node [26]. The results of our study was in agreement with those of other investigators who stated that there was no correlation between stromal CD10 expression and lymph node invasion [9,14]. Almost all patients with lymphovascular invasion were CD10 positive, except for two. Sadaka et al. and Ali et al. reported that lymphovascular invasion is significantly associated with stromal CD10 expression [28,30]. In agreement to this, our study also demonstarted a significant correlation between CD10 expression and lymphovascular invasion with a p-value of 0.006. Regarding perineural invasion, no meaningful correlation was found in this study. Some of the limitations of the current study include, a single centre study, limited sample size and the study being constrained to one geographical location. These could be a few reasons pointing to the lack of correlation of the parameters in this study as compared to the other studies seen above. Larger, multi-centre or multi-institutional studies involving a larger demography of patients could throw some light on the potential of CD10 in evaluating invasive breast carcinoma of no special type.

## Conclusions

According to previous studies, CD10 expression is seen in a high percentage of high-grade invasive breast carcinoma. However, in this study, statistical significance was found between stromal CD10 expression and lymphovascular invasion. The other clinicopathological parameters studied had no significant association, including the hormone status of the tumour. The nonsignificant results may be due to the sample size, which may not be truly representative of the trend in the general population, as well as due to geographical variations. Hence, further performing this study with a larger sample size may shed light on our hypothesis. In the future, CD10 has the potential to be used as a marker for prognosis and also for targeted therapy in breast cancer patients.
